# Phosphoinositide-3-kinase/akt - dependent signaling is required for maintenance of [Ca^2+^]_i,_*I*_Ca_, and Ca^2+^ transients in HL-1 cardiomyocytes

**DOI:** 10.1186/1423-0127-19-59

**Published:** 2012-06-20

**Authors:** Bridget M Graves, Thomas Simerly, Chuanfu Li, David L Williams, Robert Wondergem

**Affiliations:** 1 Departments of Surgery, James H. Quillen College of Medicine, East Tennessee State Universitycpr, Johnson City, TN, 37614, USA; 2 Departments of Biomedical Science, James H. Quillen College of Medicine, East Tennessee State University, Johnson City, TN, 37614, USA; 3 Department of Physiology/Biomedical Science, James H. Quillen College of Medicine, East Tennessee State University, P.O. Box 70,576, Johnson City, TN, 37614-1708, USA

**Keywords:** Calcium, Fura-2, Phosphoinositide-3-kinase/Akt, HL-1 cardiomyocytes, Whole-cell voltage clamp, Electrophysiology

## Abstract

The phosphoinositide 3-kinases (PI3K/Akt) dependent signaling pathway plays an important role in cardiac function, specifically cardiac contractility. We have reported that sepsis decreases myocardial Akt activation, which correlates with cardiac dysfunction in sepsis. We also reported that preventing sepsis induced changes in myocardial Akt activation ameliorates cardiovascular dysfunction. In this study we investigated the role of PI3K/Akt on cardiomyocyte function by examining the role of PI3K/Akt-dependent signaling on [Ca^2+^]_i_, Ca^2+^ transients and membrane Ca^2+^ current, *I*_Ca_, in cultured murine HL-1 cardiomyocytes. LY294002 (1–20 μM), a specific PI3K inhibitor, dramatically decreased HL-1 [Ca^2+^]_i_, Ca^2+^ transients and *I*_Ca_. We also examined the effect of PI3K isoform specific inhibitors, *i.e.* α (PI3-kinase α inhibitor 2; 2–8 nM); β (TGX-221; 100 nM) and γ (AS-252424; 100 nM), to determine the contribution of specific isoforms to HL-1 [Ca^2+^]_i_ regulation. Pharmacologic inhibition of each of the individual PI3K isoforms significantly decreased [Ca^2+^]_i_, and inhibited Ca^2+^ transients. Triciribine (1–20 μM), which inhibits AKT downstream of the PI3K pathway, also inhibited [Ca^2+^]_i_, and Ca^2+^ transients and *I*_Ca_. We conclude that the PI3K/Akt pathway is required for normal maintenance of [Ca^2+^]_i_ in HL-1 cardiomyocytes. Thus, myocardial PI3K/Akt-PKB signaling sustains [Ca^2+^]_i_ required for excitation-contraction coupling in cardiomyoctyes.

## Background

The phosphoinositide 3-kinases (PI3K) are a conserved family of signal transduction enzymes that are involved in regulating cellular proliferation and survival [1,2]. The PI3Ks and the downstream serine/threonine kinase Akt (also known as protein kinase B; PKB) regulate cellular activation, inflammatory responses, chemotaxis and apoptosis [1]. We [3] and others [4] have demonstrated that activation of PI3K/Akt dependent signaling attenuates the pro-inflammatory phenotype and increases survival outcome in sepsis. We have also reported that sepsis decreases myocardial Akt activation [5], which correlates with cardiac dysfunction in sepsis. In the same report, we demonstrated that preventing sepsis-induced changes in myocardial Akt activation correlates with prevention of cardiac dysfunction [5].

PI3K/Akt/PKB may play a role in cardiomyocyte calcium regulation; however, the precise mechanisms by which this occurs have not been fully elucidated. Yano and colleagues employed a transgenic mouse model over-expressing PI3K p110α in the heart [6], which resulted in increased left ventricular pressure, increased levels of L-type Ca^2+^ channels, ryanodine receptors and sarcoplasmic reticulum Ca^2+^-ATPase 2a [6]. In a subsequent report, Lu et al. demonstrated that genetic ablation of PI3K p110α resulted in reduced numbers of voltage-dependent L-type Ca^2+^ channels in isolated cardiomyocytes, reduced inward Ca^2+^ current and a defect in contractile function [7]. Taken together the results above indicate that PI3k/Akt signaling plays a critical role in normal cardiac function and in maintaining cardiac function in sepsis [5-7]. This signaling most likely involves regulation of cellular calcium.

We conducted the present study to determine whether direct inhibition of the PI3K, PI3K-specific isoforms or Akt-PKB signaling in HL-1 cardiomyocytes alters calcium regulation. HL-1 is a proliferating atrial myocyte cell line established from a subcutaneous tumor of AT-1 cells that, in turn, were derived from the atria of a mouse transgenic for the simian virus 40 large T antigen under control of the atrial natriuretic factor promoter [8,9]. These cells display spontaneous contractions in tissue culture, oscillations of [Ca^2+^_i_, and express functional L- and T-type Ca^2+^ channels [10]. HL-1 cells also express the PI3K/Akt-PKB signaling pathway, which mediates interleukin-18 induced cellular hypertrophy [11]. Herein, we report that inhibitors of PI3K/Akt-PKB decrease [Ca^2+^, diminish Ca^2+^ transients and inhibit membrane Ca^2+^ currents, *I*_Ca_, in these murine cardiomyocytes. These data indicate that PI3K/Akt-PKB is required for normal cardiomyocyte calcium regulation.

## Methods

### HL-1 cell culture

HL-1 atrial cardiomyocytes were a gift of Dr. William Claycomb (Louisiana State University Medical Center). They were grown in 5% CO_2_ at 37 °C in Claycomb media (Sigma) supplemented with batch specific 10% FBS (Sigma), 100 U/ml:100 μg/ml Penicillin/Streptomycin (Invitrogen), 0.1 mM norepinephrine (Sigma) and 2 mM L-glutamine (Invitrogen). Before culturing cells, flasks were treated overnight with 0.02% Bacto© gelatin (Fisher Scientific):0.5% Fibronectin (Invitrogen). For electrophysiologic or calcium measurements cells were plated at a density of 3X10^5^ cells/35-mm culture dish on glass cover slips (12 mm diameter), which had been flamed briefly to enhance coating and then transferred to a 35-mm culture dish where they were treated with gelatin/fibronectin overnight.

### Whole-cell voltage clamp measurements

Cells were grown for 1–2 days on 12-mm diameter glass plastic coverslips, which were transferred to an acrylic chamber (Warner, New Haven, CT) on the stage of an inverted microscope (Olympus IMT-2) equipped with Hoffman modulation contrast optics. Cells were superfused at room temperature with a standard external salt solution. Patch pipettes (3–6 MΩ in the bath solution) were fabricated from glass capillaries (1.1-1.2 mm ID, 0.2 mm wall thickness, non-heparinized micro-hematocrit capillary tubes; Fisher Scientific) with a Brown-Flaming horizontal micropipette puller (P-97, Sutter Instruments, Novato, CA). A micromanipulator (MO-202, Narishige, Tokyo) fixed to the microscope was used to position pipettes. The whole-cell patch configurations were obtained by standard patch clamp technique [12]. Voltage-clamp currents were measured with a patch clamp amplifier (Axopatch 200B, Axon Instruments, Foster City, CA) with the lowpass, Bessell filtering (−3 dB) set at 5 kHz. Signals from the patch clamp amplifier were fed into a computer via a digital interface (Digidata 1322A) and processed by Clampex 8 software (Axon Instruments). Ag/AgCl half-cells constituted the electrodes, and agar bridges (4% w v^-1^ in external solution) connected the reference electrodes to the bath solution. Series resistances were compensated following whole-cell access prior to recordings. Giga-ohm seals between pipettes and cell membranes were made with cells perfused with standard external solution. For *I*_Ca_ measurements the cells were perfused with an external solution in which an impermeable cation was substituted for Na^+^, and Ca^2+^ concentration was increased (below).

### Intracellular Ca^2+^ measurements

Cells were loaded with Fura-2/AM (TefLabs, Austin, TX) by incubating them for 30 min at room temperature (22–23 °C) with a standard external salt solution containing 2-μM Fura-2/AM. Cells were then washed with the external salt solution and incubated at 37 °C with 5% CO^2^ for 30 min in the supplemented Claycomb media. The coverslip was transferred to an acrylic chamber (Warner Instr. Co., Hamden, CT) on the microscope stage and washed with the external salt solution for 5 minutes before measurements. Temperature was maintained throughout measurements at 37 °C by a stage/inline temperature controller (Warner Instr. Co., Hamden, CT) Fluorescence was measured with an imaging system consisting of a xenon fiberoptic light source (Perkin-Elmer, Waltham, MA), a filter wheel and a Basler A311F VGA Camera connected to an Olympus IX71inverted fluorescence microscope. The filter wheel and data acquisition were controlled by the InCyte2 software (v. 5.29; Intracellular Imaging, Cincinnati, OH). [Ca^2+^] was determined by interpolation from a standard curve generated by Ca^2+^ calibration buffer kit #2 (Molecular Probes @ Life Technologies, Grand Island, NY) and Fura-2/K_5_-salt. After correction for the individual background fluorescence, the ratio of the fluorescence at both excitation wavelengths (F340/F380) was monitored simultaneously in 30–40 cells, identified by their fluorescence within a single view field. Images were collected every 3.3 s. Each slide was perfused with standard external salt solution for 6 min for control measurements, followed by 10 min with the experimental solution. At 16 min, the slide was washed with standard external salt solution for 5 min, and at 21 min data collection was stopped. Data was then exported to MS Excel and graphed using Origin 7.0 (OriginLab Corp., Northampton, MA) and Sigmaplot 11.0 (Systat Software, Inc., Chicago, IL). For statistical analyses, average [Ca^2+^]_i_ from 25–40 cells within a microscopic field were obtained during the control period of 1–5 min from each of 5 separate HL-1 cell preparations. These averages were then compiled to obtain average control values (n = 5), and comparisons were made on data collected similarly from the same microscopic fields 15 minutes after experimental additions. Statistical differences between control and experimental values were established at p < 0.05 (Student’s paired *T*-test).

### Solutions and chemicals

Standard external salt solution contained (mM): NaCl 150, KCl 6, MgCl_2_ 1, CaCl_2_ 1.5, N-2-Hydroxyethylpiperazine-N’-2-ethanesulfonic acid (HEPES) 10, glucose 10 (pH adjusted to 7.41 with NaOH). Pipette solution contained (mM): potassium aspartate 120, Na_2_GTP 0.4, Na_2_ATP 5, MgCl_2_ 1, EGTA 5, CaCl_2_ 0.1, HEPES 10 (pH adjusted to 7.2 with KOH). For whole-cell voltage clamp measurements of membrane Ca^2+^ currents external NaCl was substituted with (mM) *n*-methy-*D*-gluamine chloride (NMDG^+^) 150, and CaCl_2_ was increased to 5 to maximize Ca^2+^ current. All solution constituents were obtained from Sigma/Aldrich, St. Louis, MO.

LY 294002 was obtained from Alomone Labs, LTD, Jerusalem, Israel. The PI3 kinase isoform inhibitors: PI3-kinase α inhibitor 2, TGX-221 β inhibitor, AS-252424 γ isoform inhibitor; and the AKT inhibitor, Triciribine were obtained from Cayman Chemical, Ann Arbor, MI. The dosages selected for the various inhibitors were based on the literature and the manufacturer’s instructions. All inhibitors were dissolved in DMSO in stock solutions and then diluted to final concentration. The highest final concentration of DMSO by this approach was 0.24% DMSO.

## Results

### Pharmacologic inhibition of PI3K significantly reduces [Ca^2+^]_i_, and Ca^2+^ transients in HL-1 cardiomyocytes

**Figure 1:**
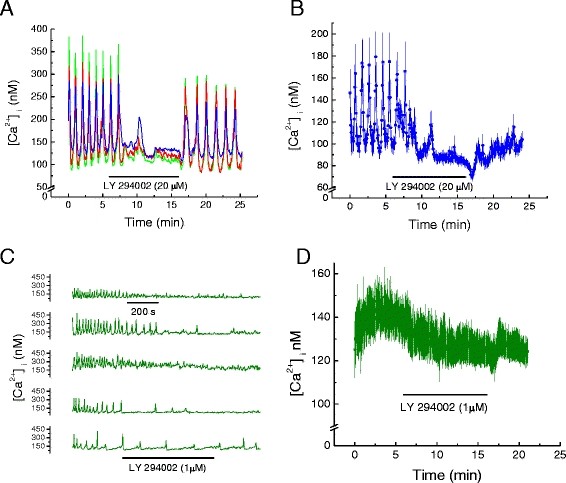
**Phosphoinositide-3-kinase (PI3K) inhibition decreases intracellular Ca**^**2+**^**, [Ca**^**2+**^**]**_**i**_**, in HL-1 cell mouse cardiomyocytes.** ***A***. Effect of LY294002 (20 μM) on oscillations of [Ca^2+^]_i_ in three HL-1 cells showing synchronous oscillations of [Ca^2+^]_i_. ***B***. Effect of LY294002 (20 μM) on average [Ca^2+^]_i_ in cells displaying oscillating and non-oscillating [Ca^2+^]_i_ (mean ± SEM; n = 37 cells). ***C.*** Effect of LY294002 (1 μM) on [Ca^2+^]_i_ oscillations in five representative HL-1 cells. Time base applies to all traces. ***D***. Effect of LY294002 (1 μM) on average [Ca^2+^]_i_ in cells displaying oscillating and non-oscillating [Ca^2+^]_i_ (mean ± SEM; n = 5, each an average of 25 to 40 cells as shown in C).

Action potentials and corresponding spontaneous transients in intracellular Ca^2+^, [Ca^2+^_i_, occur in approximately 40% of non-confluent immortalized mouse HL-1 cardiomyocytes [13,14]. Synchronous Ca^2+^ transients in three such cells are shown in Figure 1A. Perfusing the cells with LY 294002 (20 μM), a potent inhibitor of phosphoinositde-3-kinases (PI3Ks), inhibited Ca^2+^ transients within 2 minutes, and this effect was partially reversed on washout. When all cells within a microscopic field (n = 37), *i.e.* those showing Ca^2+^ transients and those without transients, were included in the computation of mean [Ca^2+^_i_, the Ca^2+^ transients again were evident, but the averaging reduced their magnitude, Figure 1B. LY 294002 again abolished the Ca^2+^ transients and diminished total [Ca^2+^_i_, Figure 1B. Washout restored total [Ca^2+^_i_, but the Ca^2+^ transients were no longer apparent, except for partial restoration in 3 cells out of the 10 of 37 cells showing Ca^2+^ transients (results not shown). LY 294002 at 1 μM also inhibited Ca^2+^ transients with some restoration on washout, Figure 1C. LY 294002 at 1 μM also significantly reduced total [Ca^2+^_i_, Table 1, with modest but insignificant reversal on washout within 5 minutes, Figure 1D. Surprisingly, 10-μM LY 294002 inhibition was insignificant. We attribute this inconsistency to the variation in differentiated phenotype among the population of HL-1 cells within a microscopic field. The dynamic response of [Ca^2+^_i_ depends on Ca^2+^ oscillations [14], which in turn depend on the *I*_f_, whose phenotype varies in these cells [13] .

**Table 1:** **Effect of phosphoinositide-3-kinase inhibitor LY 294002 on intracellular Ca**^**2+**^**concentration recorded 15 minutes after addition of respective inhibitors**

**Agent** **(Concentration)** **Target**	**Control** **[Ca**^**2+**^**]**_**i**_**, (nM)**	**Inhibitor (15 min)** **[Ca**^**2+**^**]**_**i**_**, (nM)**	***P*** **value for difference**
LY 294002 (1 μM) PI3K	141.6 ± 10.1	124.9 ± 6.9	<0.03
LY 294002 (10 μM) PI3K	160.6 ± 24.4	134.8 ± 12.0	=0.29
LY 294002 (20 μM) PI3K	132.6 ± 5.2	117.1 ± 6.1	<0.003

Mean ± SEM (n = 3 or 5 experiments each, with each experiment comprising the average [Ca^2+^] of 25 to 40 cells).

### Inhibition of PI3K isoforms and akt significantly reduces [Ca^2+^]_i_, I_Ca_ and Ca^2+^ transients in HL-1 cardiomyocytes

**Figure 2:**
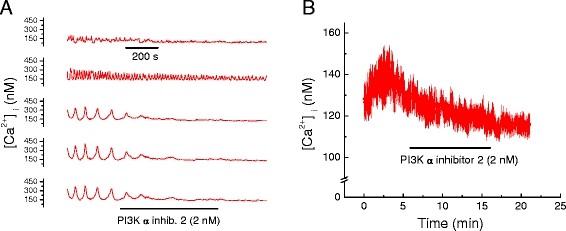
**Pharmacologic inhibition of phosphoinositide-3-kinase (PI3K) isoform α inhibitor decreased Ca**^**2+**^**, [Ca**^**2+**^**]**_**i**_**, in HL-1 cell mouse cardiomyocytes.** ***A.*** Effect of PI3K α inhibitor 2 (2 nM) on [Ca^2+^]_i_ oscillations in five representative HL-1 cells. Time base applies to all traces. ***B.*** Effect of PI3K α inhibitor 2 (2 nM) on average [Ca^2+^]_i_ in cells displaying oscillating and non-oscillating [Ca^2+^]_i_ (mean ± SEM; n = 5, each an average of 25 to 40 cells as shown in A).

**Figure 3:**
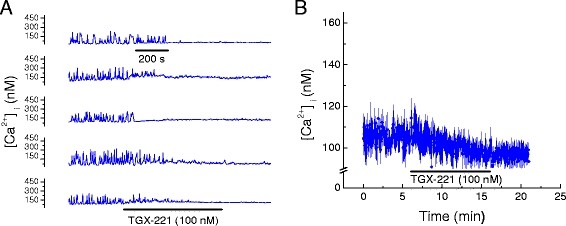
**Pharmacologic inhibition of phosphoinositide-3-kinase (PI3K) isoform β decreased Ca**^**2+**^**, [Ca**^**2+**^**]**_**i**_**, in HL-1 cell mouse cardiomyocytes.** ***A***. Effect of TGX-221 (100 nM) on [Ca^2+^]_i_ oscillations in five representative HL-1 cells. Time base applies to all traces. ***B***. Effect of TGX-221 (100 nM) on average [Ca^2+^]_i_ in cells displaying oscillating and non-oscillating [Ca^2+^]_i_ (mean ± SEM; n = 5, each an average of 25 to 40 cells as shown in A).

**Figure 4:**
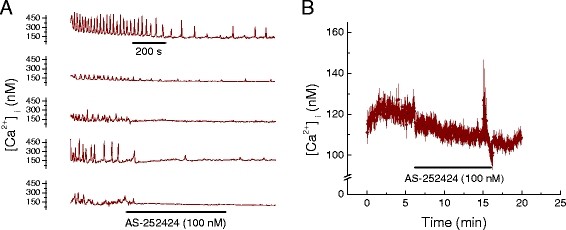
**Pharmacologic inhibition of phosphoinositide-3-kinase (PI3K) isoform γ decreases Ca**^**2+**^**, [Ca**^**2+**^**]**_**i**_**, in HL-1 cell mouse cardiomyocytes.** ***A***. Effect of AS-252424 (100 nM) on [Ca^2+^]_i_ oscillations in five representative HL-1 cells. Time base applies to all traces. ***B***. Effect of AS-252424 (100 nM) on average [Ca^2+^]_i_ in cells displaying oscillating and non-oscillating [Ca^2+^]_i_ (mean ± SEM; n = 5, each an average of 25 to 40 cells as shown in A.

**Figure 5:**
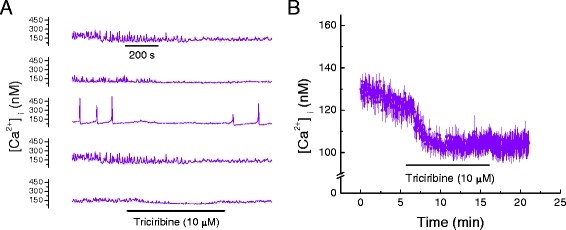
**Akt-PKB inhibition with triciribine decreases Ca**^**2+**^**, [Ca**^**2+**^**]**_**i**_**, in HL-1 cell mouse cardiomyocytes.** ***A***. Effect of triciribine (10 μM) on [Ca^2+^]_i_ oscillations in five representative HL-1 cells. Time base applies to all traces. ***B***. Effect of triciribine (10 μM) on average [Ca^2+^]_i_ in cells displaying oscillating and non-oscillating [Ca^2+^]_i_ (mean ± SEM; n = 5, each an average of 25 to 40 cells as shown in A).

Considering that LY 294002 is a broad spectrum inhibitor of PI3Ks and binds to various targets [15], we performed measurements to determine whether inhibitors of specific PI3K isoforms (*i.e.* α, β and γ) have similar effects on Ca^2+^ transients and total [Ca^2+^_i_. PI3-kinase α inhibitor 2 (2 nM) abolished Ca^2+^ transients in HL-1 cells within 3 to 4 min, Figure 2A, with no reversal on washout. It also significantly reduced total HL-1 [Ca^2+^_i_, Table 2 and Figure 2B. Identical effects were obtained for the PI3K β inhibitor (TGX-221, 100 nM), Figure 3A & 3B and Table 3, as well as for the PI3K γ inhibitor (AS-252424, 100 nM), Figure 4A & 4B and Table 3. A major downstream target of PI3K is Akt/PKB [16]. Therefore, we pharmacologically inhibited Akt in order to determine if the effect of PI3K on myocardial [Ca^2+^_i_ is mediated via Akt. Triciribine (10 μM), a specific inhibitor of Akt, also inhibited Ca^2+^ transients in HL-1 cells with modest reversal of this inhibition on washout, Figure 5A. Triciribine also significantly decreased HL-1 cell total [Ca^2+^_i_, and this did not reverse on washout, Table 4 and Figure 5B. DMSO (0.24%), the diluent used for these inhibitors, had no effect on [Ca^2+^_i_ = 125.3 ± 7.2 nM compared with Control [Ca^2+^_i_ = 131.6 ± 7.9 nM (p = 0.18; n = 5).

**Table 2:** **Effect of phosphoinositide-3-kinase α inhibitor on intracellular Ca**^**2+**^**concentration recorded 15 minutes after addition of respective inhibitors**

**Agent** **(Concentration) Target**	**Control** **[Ca**^**2+**^**]**_**i**_**, (nM)**	**Inhibitor (15 min)** **[Ca**^**2+**^**]**_**i**_**, (nM)**	***P*** **value for difference**
PI3K α inhibitor 2 (2 nM) α	136.4 ± 8.6	118.3 ± 5.9	<0.02
PI3K α inhibitor 2 (4 nM) α	114.9 ± 4.3	102.1 ± 2.5	<0.01
PI3K α inhibitor 2 (8 nM) α	131.7 ± 9.0	110.5 ± 4.7	=0.06

Mean ± SEM (n = 5 experiments each, with each experiment comprising the average [Ca^2+^] of 25 to 40 cells).

**Table 3:** **Effect of phosphoinositide-3-kinases β and γ inhibitors on intracellular Ca**^**2+**^**concentration recorded 15 minutes after addition of respective inhibitors**

**Agent** **(Concentration) Target**	**Control** **[Ca**^**2+**^**]**_**i**_**, (nM)**	**Inhibitor (15 min)** **[Ca**^**2+**^**]**_**i**_**, (nM)**	***P*** **value for difference**
TGX-221 (100 nM) β	105.5 ± 5.0	96.6 ± 4.8	<0.03
AS-252424 (100 nM) γ	121.8 ± 4.3	111.9 ± 6.0	<0.03

Mean ± SEM (n = 5 experiments each, with each experiment comprising the average [Ca^2+^] of 25 to 40 cells).

**Table 4:** **Effect of Akt-PKB inhibitor on intracellular Ca**^**2+**^**concentration recorded 15 minutes after addition of respective inhibitors**

**Agent** **(Concentration) Target**	**Control** **[Ca**^**2+**^**]**_**i**_**, (nM)**	**Inhibitor (15 min)** **[Ca**^**2+**^**]**_**i**_**, (nM)**	***P*** **value for difference**
Triciribine (1 μM) Akt-PKB	177.4 ± 5.2	157.7 ± 1.9	<0.05
Triciribine (10 μM) Akt-PKB	125.7 ± 4.4	104.6 ± 4.8	<0.03
Triciribine (20 μM) Akt-PKB	216.8 ± 4.1	203.9 ± 1.4	=0.13

Mean ± SEM (n = 3 or 5 experiments each, with each experiment comprising the average [Ca^2+^] of 25 to 40 cells).

### Pharmacologic inhibition of PI3K and Akt significantly reduces I_Ca_

**Figure 6:**
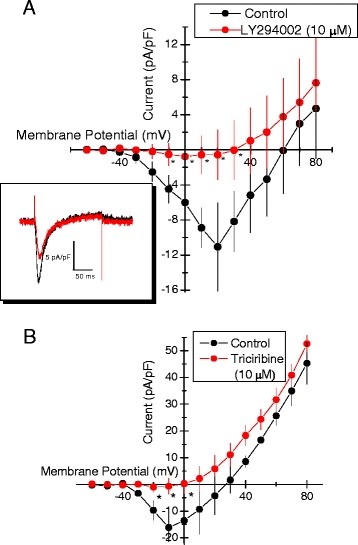
**Pharmacologic inhibition of phosphoinositide-3-kinase (PI3K) and Akt-PKB decrease Ca**^**2+**^**current in HL-1 cell mouse cardiomyocytes.** ***A***. Effect of LY294002 (10 μM) on I/V plot of Ca^2+^ current compared with control (mean ± SEM; n = 3). Holding potential = −50 mV. *Inset*. Overlay of currents recorded in same cell ***before*** and ***after*** LY294002 (10 μM) during a voltage step from −50 mV (HP) to 0 mV. Baseline current at −50 mV prior to voltage step to 0 mV was 0 nA. ***B***. Effect of triciribine (10 μM) on Ca^2+^ current compared with control (mean ± SEM; n = 3). Holding potential = −50 mV.

As an initial step to determine whether the effects by inhibitors of PI3Ks and of Akt on Ca^2+^ transients and total [Ca^2+^]_i_ resulted from inhibition of membrane Ca^2+^ channels, we determined the effect of LY 294002 and triciribine on membrane Ca^2+^ currents in HL-1 cells. For these measurements the pipette-membrane giga-ohm seal and whole-cell access were obtained with cells perfused with standard external solution. Once achieved, the external solution was exchanged to one in which Na^+^ was substituted with NMDG^+^ and [Ca^2+^]_o_ increased from 1.5 to 5 mM. Following 5-min equilibration, voltage clamp step protocols were performed to generate current–voltage plots (I/V plots, Figure 6) obtained at maximal inward current, Figure 6A*inset*, under control conditions and following five minutes of an inhibitor of either PI3Ks or Akt. The holding potential throughout these measurements was −50 mV. Depolarizing voltage steps activated inward current at ~ −40 mV, with maximal inward current occurring with depolarizations ranging from −10 to 20 mV, Figure 6A and 6B. The voltage-activated inward currents were inhibited completely by perfusing the cells for 5 min with either LY 294002 (10 μM), Figure 6A, or with triciribine (10 μM), Figure 6B. Inward currents also were completely abolished by perfusing the cells with external solution in which NMDG^+^ substituted for both Na^+^ and Ca^2+^ (results not shown).

## Discussion

These findings show that PI3K/Akt-PKB signaling pathways play a significant role in regulating intracellular Ca^2+^ in HL-1 cells, which constitute a murine-derived, immortalized cell line with phenotypes like those of adult cardiomyocytes [8,9]. We found that LY 294002, a specific inhibitor of PI3K, as well as specific inhibitors of each of the PI3K isoforms, *i.e.* α, β and γ catalytic PI3K subunits, and an inhibitor of Akt/PKB, significantly decreased [Ca^2+^_i_ and abolished Ca^2+^ transients or oscillations. Moreover, inhibition of PI3K/Akt-PKB signaling pathways abolished inward Ca^2+^ current in the HL-1 cells, which likely results from L-type Ca^2+^ channels in HL-1 cells.

Taken together we conclude that the PI3K/Akt-PKB signaling pathway plays a role in sustaining the voltage-activated Ca^2+^ current contributing to the HL-1 cell action potential. Catalucci et al. [17] have shown that Akt-dependent phosphorylation of Ca_v_β2, the chaperone of the L-type Ca^2+^ channel pore-forming subunit, Ca_v_α1, antagonizes Ca_v_α1 degradation and, as such, stabilizes the functional channel in the plasma membrane. Inward Ca^2+^ currents from action potential, via voltage-activated membrane Ca^2+^ channels, induce Ca^2+^ release from the sarcoplasmic reticulum [18,19], which accounts for excitation-contraction coupling in cardiomyocytes [20].

We observed a two- to five-minute delay for various PIK3/Akt-PKB inhibitors to reduce Ca^2+^ transients, [Ca^2+^_i_ and *I*_Ca._ This is consistent with a time course for the manifestation of inhibition of an enzymatic signaling cascade. We conclude also that this delay is inconsistent with a direct inhibition of membrane Ca^2+^ channels by the various inhibitors, which most likely would occur faster. The marked reduction of *I*_Ca_ by PI3K/Akt-PKB inhibitors likely results from diminution of L-type *I*_Ca_. We cannot rule out involvement of T-type *I*_Ca_ since both are expressed in HL-1 cells [10]. However, based upon our holding potential of −50 mV compared with the more electronegative activating voltages for T-type Ca^2+^ channels [10] and the relatively extended time course of our *I*_Ca_, the effects measured here are likely those of L-type *I*_Ca_. Finally, we conclude that the large outward currents seen in the I/V plots at potentials >30 mV result from K^+^ currents whose magnitude we have found to vary considerably among HL-1 cells in non-confluent culture (Wondergem, *unpublished observations*).

These findings also have implications for our understanding of the role of PI3K/Akt-PKB signaling in disease. As noted above, we have reported that sepsis results in decreased activation of the PI3K/Akt pathway in the myocardium [5]. We have also discovered that constitutive up regulation of PI3K p110α in the myocardium prevents sepsis induced cardiac dysfunction and improves survival outcome in septic mice (Li, Williams and colleagues, *unpublished observations*). Although PI3K/Akt-PKB inhibition in septic mice undoubtedly leads to increased cytokine production in these animals [3], the present findings also indicate that PI3K/Akt-PKB inhibition directly decreases availability of Ca^2+^ in the mouse cardiomyocytes. Consistent with this conclusion are the reports that ventricular myocytes obtained from endotoxemic guinea-pigs [21] and septic pigs [22] show marked reduction in L-type calcium current; whereas, Akt/PKB overexpression in transgenic mice results in cardiac hypertrophy, increased amplitude of Ca^2+^ transients and enhanced L-type membrane Ca^2+^ currents [23]. Lipopolysaccharide treatment of rats also leads to arrhythmogenesis attributable to reduced mRNA levels encoding for L-type Ca^2+^ subunits [24]. We reported that LPS directly reduces Ca^2+^ transients in HL-1 cells; however, LPS has no direct effect on L-type Ca^2+^ currents in these cells, acting instead to reduce the funny current, *I*_f_, [14] as also shown by others [25]. Thus, to whatever extent sepsis reduces cardiomyocyte [Ca^2+^_i_ and Ca^2+^ transients by inhibition of PI3K/Akt-PKB, elevated cytokines most likely effect these reductions and not LPS directly. On the other hand, the present findings suggest that the amelioration of sepsis and endotoxemia by preconditioning [26] or ischemia [27] may result from upregulation of the PK3/Akt-PKB signaling pathways [3,28-32], which directly increases [Ca^2+^_i_ available for excitation-contraction coupling in cardiomyocytes.

All PI3K/Akt-PKB inhibitors used in these experiments inhibited Ca^2+^ transients and significantly decreased [Ca^2+^_i_; however, we cannot attribute the importance of one inhibitor over and against that of the others. Similar inhibition Ca^2+^ transients and [Ca^2+^_i_ by LY294002 at either 20- or 1-μM rules out toxicity by the drug. Still, there is considerable variability among HL-1 cells regarding the amplitude and rate of Ca^2+^ transients, as well as [Ca^2+^_i_. We attribute this to variation in differentiated phenotype of HL-1 cells 1–2 days after passage. While paired comparisons of the results between control and experimental conditions clearly demonstrate effects of these inhibitors, the variation of Ca^2+^ transients and [Ca^2+^_i_ among cells precludes analyses of the comparative effectiveness of the inhibitors. We cannot readily account for Ca^2+^ variability except to point out that these measurements were made on single HL-1 cells or on small islands of multiple cells as compared with confluent cell monolayers. Usually HL-1 cells require cell confluence following 3–4 days in culture to fully establish the contracting phenotype [8,13], which also suggests variable expression of ion channels needed for this rhythmicity. As noted, we have observed marked variation in the magnitude of outward K^+^ currents in HL-1 cells under these conditions. Indeed, we have been able to elicit robust Ca^2+^ transients in otherwise quiescent cells by perfusing the cells with an inhibitor of the delayed-rectifier K^+^ channels (E-4031, 10 μM; Graves and Wondergem, *unpublished observations*); which are prevalent in HL-1 cells [8,33]. Thus, variation among HL-1 cells in the strength of repolarizing K^+^ current during action potentials or in cells at rest may account for the different rates and amplitudes of Ca^2+^ transients as well as [Ca^2+^_i_.

## Conclusions

In sum, we have found that inhibitors of the PI3K/Akt signaling cascade decrease total [Ca^2+^]_i_, intracellular Ca^2+^ transients and membrane *I*_Ca_ in a murine, immortalized cardiomyocyte cell line, HL-1 cells. These data demonstrate that PI3K/Akt dependent signaling is required for normal Ca^2+^ metabolism in murine cardiomyocytes. This extends our knowledge of the role of PI3K/Akt signaling in cardiovascular homeostasis. We conclude that maintaining myocardial PI3K/Akt signaling is essential for cardiomyocyte function in the presence and absence of disease.

## Abbreviations

PI3K, Phosphoinositide-3-kinase; Akt, Protein kinase B; HL-1 cells, Proliferating atrial myocytes established from a tumor of AT-1 cells that, in turn, were derived from the atria of a mouse transgenic for the simian virus 40 large T antigen under control of the atrial natriuretic factor promoter; [Ca2+]i, Intracellular calcium ion; ATP, Adenosine triphosphate; GTP, Guanosine triphosphate; HEPES, N-2-Hydroxyethylpiperazine-N’-2-ethanesulfonic acid; ICa, Membrane calcium current; FBS, Fetal bovine serum; EGTA, Ethylene glycol-bis(2-aminoethylether)-N,N,N′,N′-tetraacetic acid; NMDG, n-methy-D-gluamine; I/V, Current–voltage; L-type, Long-lasting; T-type, Transient; If, Funny current.

## Competing interests

The authors declare that they have no competing interests.

## Authors’ contributions

BMG wrote the first draft of the manuscript, performed all Ca^2+^ measurements and analyzed data; TS analyzed data and contributed to writing and editing of the manuscript; CL contributed to the experimental design and writing of the manuscript; DLW designed experiments and wrote and revised the manuscript; RW performed all electrophysiology and wrote and edited the manuscript. All authors read and approved the final manuscript.
